# Performance of a malaria microscopy image analysis slide reading device

**DOI:** 10.1186/1475-2875-11-155

**Published:** 2012-05-06

**Authors:** William R Prescott, Robert G Jordan, Martin P Grobusch, Vernon M Chinchilli, Immo Kleinschmidt, Joseph Borovsky, Mark Plaskow, Miguel Torrez, Maximo Mico, Christopher Schwabe

**Affiliations:** 1 Hydas World Health, Hershey, PA, 17033, USA; 2 Department of Infectious Diseases, Center for Tropical Medicine and Travel Medicine, Tropical Medicine and AIDS, Division of Internal Medicine, Academic Medical Centre, University of Amsterdam, The Netherlands and the Institute of Tropical Medicine, University of Tübingen, Tübinen, Germany; 3 Department of Public Health Sciences, Penn State Hershey College of Medicine, Hershey, PA, 17033, USA; 4 MRC Tropical Epidemiology Group, London School of Hygiene and Tropical Medicine, Keppel Street, London, WC1E 7HT, UK; 5 World Health Technologies, 4101 Audley Rd, New Albany, OH, 43054, USA; 6 Medical Care Development International, 8401 Colesville Rd., Suite 425, Silver Spring, MD, 20910, USA; 7 National Malaria Control Programme, Ministry of Health and Social Welfare, Malabo, Equatorial Guinea

**Keywords:** Malaria, Diagnosis, Image analysis, Microscopy

## Abstract

**Background:**

Viewing Plasmodium in Romanovsky-stained blood has long been considered the gold standard for diagnosis and a cornerstone in management of the disease. This method however, requires a subjective evaluation by trained, experienced diagnosticians and establishing proficiency of diagnosis is fraught with many challenges. Reported here is an evaluation of a diagnostic system (a “device” consisting of a microscope, a scanner, and a computer algorithm) that evaluates scanned images of standard Giemsa-stained slides and reports species and parasitaemia.

**Methods:**

The device was challenged with two independent tests: a 55 slide, expert slide reading test the composition of which has been published by the World Health Organization (“WHO55” test), and a second test in which slides were made from a sample of consenting subjects participating in a malaria incidence survey conducted in Equatorial Guinea (EGMIS test). These subjects’ blood was tested by malaria RDT as well as having the blood smear diagnosis unequivocally determined by a worldwide panel of a minimum of six reference microscopists. Only slides with unequivocal microscopic diagnoses were used for the device challenge, n = 119.

**Results:**

On the WHO55 test, the device scored a “Level 4” using the WHO published grading scheme. Broken down by more traditional analysis parameters this result was translated to 89% and 70% sensitivity and specificity, respectively. Species were correctly identified in 61% of the slides and the quantification of parasites fell within acceptable range of the validated parasitaemia in 10% of the cases. On the EGMIS test it scored 100% and 94% sensitivity/specificity, with 64% of the species correct and 45% of the parasitaemia within an acceptable range. A pooled analysis of the 174 slides used for both tests resulted in an overall 92% sensitivity and 90% specificity with 61% species and 19% quantifications correct.

**Conclusions:**

In its current manifestation, the device performs at a level comparable to that of many human slide readers. Because its use requires minimal additional equipment and it uses standard stained slides as starting material, its widespread adoption may eliminate the current uncertainty about the quality of microscopic diagnoses worldwide.

## Background

Despite tremendous recent gains, the World Health Organization (WHO) still reports over 225 million cases and nearly 800,000 deaths in its most recent report [1]. In addition to vector control through indoor residual spraying and insecticide treated bed nets, and improved treatment based on artemisinin combination therapy, prompt and accurate diagnosis is a critically important additional factor in fighting this disease. For comparisons of malaria diagnostic modalities, the microscopic examination of Romanowsky-stained smears is widely considered to be the clinical “gold standard” [2,3]. Even 100 years after Ronald Ross’ Nobel Prize for his microscopy-based work on malaria, it remains the only diagnostic method in which: a) the parasite is visualized, b) the result is both qualitative and quantitative, c) prognostic factors such as the presence of *Plasmodia* gametocytes or the rate of haemozoin-containing macrophages, can be assessed [3], and d) alternative/additional infections such as blood-dwelling helminthes or spirochetes can be diagnosed. Indeed, numerous publications over the past 10–15 years that have sought to evaluate new malaria diagnostics, for example immunochromatographic strips, known as malaria rapid diagnostic tests (RDTs), polymerase chain reaction (PCR), or qualitative buffy coat microscopy (QBC) have done so by comparing their results to those of “expert” microscopy [4].

“Expert microscopy” as an entity, however, is not always consistent with respect to a number of aspects that can have a direct effect on the results obtained. An important example of this inconsistency is in the length of time a slide is reviewed. A review of 88 studies demonstrated as much as 20-fold variations in the measures of the time spent reviewing a slide such as high power fields or leucocytes amassed [5]. The sequelae of such variability are inaccuracies of diagnosis which can affect not only a single patient, but can also have significant deleterious effects in clinical trials investigating new drugs or vaccines [6].

Malaria microscopy is a skill which requires considerable training, experience and practice to achieve and maintain proficiency. Even to make the measurement of that proficiency consistent and fair is a daunting task. While examining a blood film, the well-trained microscopist looks at and integrates other aspects of the haematology being examined into the diagnosis. A relevant example is the identification of *Mansonella perstans* filariasis in several of the patients involved in this study. They were found and identified by the microscopists, but were undetectable by malaria RDTs. To ensure quality malaria diagnosis by microscope, significant challenges are involved not only in pre-service training, but also in maintaining the proficiency of human microscopists, both in endemic settings and as well as in the parts of the world with imported malaria.

Given these challenges and inherent variability, there has long been a quest for alternative, if not less subjective malaria diagnostic methods, and a wealth of tools have been developed, some even experimentally evaluated. Recently the prospect of using scanning technology and a computer algorithm to analyze images captured from the microscopic examination of stained blood smears to find, identify and quantify malaria parasites has emerged. In a review in 2008 John Frean first pointed out the suitability of image analysis for enumerating malaria parasites [7]. A year later he reported on software used to count parasites in individually captured microscope images, a study which showed good agreement between the program and human counts made from the same images. The correlations were especially good at high counts (>100,000 parasites/ μL) but became “poor” at concentrations below 6 parasites/image (presumably, below approximately 20,000 parasites/ μL of blood) and were generally about 27% higher than that recorded by humans reading the slides with a microscope [8].

Purwar et al. [9] reported on a screening tool which uses only thin film images and is “designed to be overly sensitive so that no true cases of malaria are missed.” Likewise, Proudfoot et al. describes a diagnostic aid [10] using commercially available software. In terms of automated diagnosis, he was able to produce a composite in which the RBC images were “ranked” based on the probability that they were infected. An in-depth review of the area of thin film malaria detection was provided by Tek et al. in 2009 [11].

This paper, reports on the evaluation of a similar algorithm, however one which uses both thick and/or thin blood films and is coupled with an automated scanner. The coupling of the scanner with a PC comprises a completely automated system which is referred to here as a “device.” This device was evaluated using slides in accordance with WHO published testing standards and under field conditions within the Bioko Island Malaria Control Project (BIMCP) in Equatorial Guinea.

## Methods

**Figure 1:**
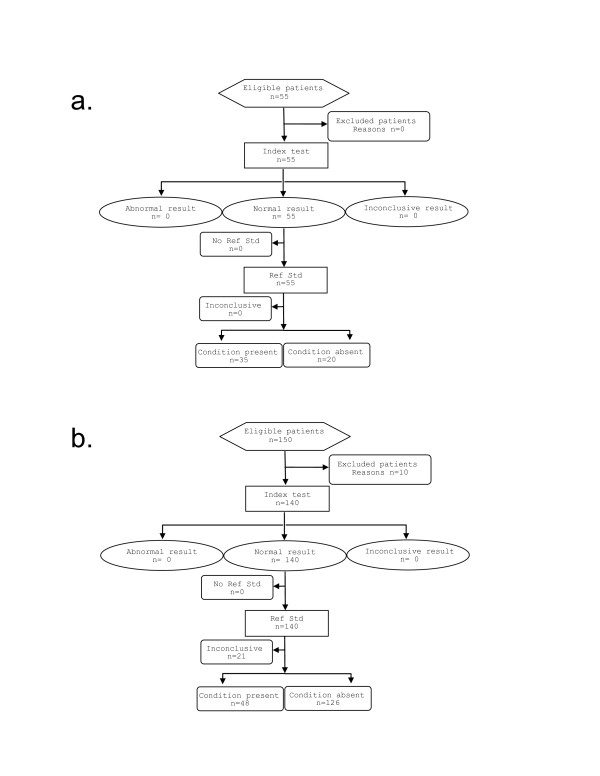
STARDS flow diagram for the WHO 55 test (a) and for the EGMIS test (b).

The device was challenged with two independent tests. The first was a 55 slide expert microscopy test using validated archived slides (WHO55 test). The second challenge came from 140 slides garnered from a malaria prevalence survey, the Equatorial Guinea Malaria Indicator Survey (EGMIS). The two tests are reported both separately and as pooled data. In Figures 1 a and b, the flow diagram suggested by the Standards for Reporting of Diagnostic Accuracy (STARD Initiative)[12] is shown for each of the two tests.

### World Health Technology (WHT) automated scanning

The WHT software technology uses digital images made from standard Giemsa or Field’s stained microscope slides. Either digital microscopes or imaging scanners may be used to acquire the images that are stored and subsequently serve as the input for the algorithm to locate, identify and count the parasites. Localization, recognition and enumeration of the salient constituents of the scans (parasites and leucocytes) are based on pattern, color and shape recognition of parasites in red blood cells (RBCs) of a thin film and/or parasites that remain in a thick film after lysis of RBCs. The algorithms were developed with the help of pathologists experienced in malaria, and are able to distinguish malaria parasites from other blood constituents and artifacts. As the database of such images increases, the software will be enhanced to be more proficient at distinguishing interferences and anomalies from parasites, thereby improving the accuracy of the diagnosis.

**Figure 2:**
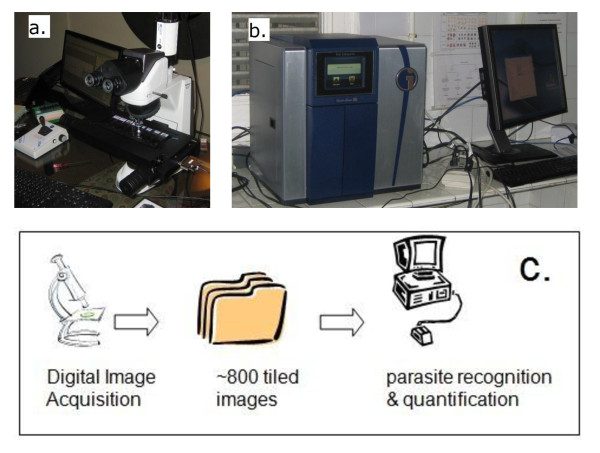
** The WHT Automated Malaria slide reading system.a)** Doctors’ Choice® scanner; **b)** IScan Coreo Gold® scanner, on site in Malabo, Equatorial Guinea; **c)** diagrammatic representation of the process.

To use the WHT system, a slide is placed in the scanner (with an automated scanner many slides can be placed at one time), and the scanner captures images at the selected magnification. Two scanners were used for this study, IScan Coreo Gold® (Vantana Corp., Sunnyvale, CA) and Doctor’s Choice®(Intracellular, Cincinnati, Ohio), a custom portable device made to WHT specifications (Figure 2, a and b). The functions of both scanners were very similar to include speed of scanning, color and shade adjustment as well as magnification and image clarity. The primary difference between them is that the IScan Coreo Gold® is capable of handling 160 slides while the Doctor’s Choice® is limited to 8 at one time. The scanner first provides an image of the entire slide to allow the operator, with the help of the scanner software, to mark areas of interest on the slide. A square shape is applied to encompass most of the round thick smear and a rectangle to include the feathered edge of the thin smear. Since all slides used in this study were made using a template, the smears were all in the same position on each and the same boundaries were used for all of the scans. The only additional operator interaction with the process is to identify autofocus points for the scanner. Approximately 8–10 autofocus points are used for each thick and thin film. Since the scanned area is defined, the greater the magnification the longer the scanning time. For this study slides were scanned using a 40x objective which took approximately five minutes per slide. At this magnification, the scanner creates approximately 800 tiled digital images (800 high power fields) of the thick film area, which are used as the input data for the diagnostic algorithm. Upon being captured, they are immediately input to the WHT software and results are obtained for the 800 images in less than a minute (2, c).

The software performs three major operations: pre-scan, removal of artifacts, and a full software analysis.

(i) Pre-scan: the software compares all the images against a set of “tight criteria” which are parameters designed to find the malaria parasites, if present. In order to be deemed positive, a pre-set threshold level of items matching these tight criteria must be found. If the threshold criterion is met, the software executes the next step.

(ii) Removal of artifacts in the scanned digital image: blood components that may be confused with malaria parasites, including platelets, stain crystals and other interfering images are digitally “removed.” An iterated threshold is used that defines the parameters used to form a binary image. Pixel intensity is used as a part of this iterative threshold, which then separates foreground from background and therefore, cellular blood constituents from artifact. Despite ignoring these potential confounds in the subsequent processing, the software does not alter the originally acquired scan. At the end of this operation the “cleaned” scan is now ready for the final step.

(iii) Full software scan: the resulting ‘images-of-interest’ are compared to a wide range of parameters that describe the parasite by employing a databased library. It is the augmentation of this library that will allow the performance that has been recorded in this study to be improved over time as relevant shapes are added. This is the step that identifies the cellular constituents of interest, whether in thick or thin film and therefore allows the counting of all the parasites and white blood cells (WBCs), and calculates the infection density. Density is determined by establishing a ratio of parasites to the observed number of white blood cells (WBC). Because WBCs and parasites are not homogeneously distributed across the slide [13], these two elements are totaled across all 800 tiles and the calculation done on the resulting sums and rounded to the nearest multiple of 10. Therefore, by assuming a standard of 8,000 WBCs per microliter (μL), the software is able to express the parasitaemia in units of parasite/μL of blood in much the same way that human readers quantify the parasitaemia of a slide [14], pp67-68. For the case in which a patient’s complete blood count (CBC) is known however, the actual WBC/μL value may be used instead of the 8,000 approximation.

In a very comparable sequence of events to that carried out by human readers, the scanner collects images from the thin film in order to identify the parasite species after processing the images collected from the thick film. Unlike the thick film, the thin film is methanol fixed during the preparation of the slide. In the thin film, the intact RBCs are visible and the morphology of parasites *in situ* may be “seen” and evaluated by the algorithm. Therefore visual cues (“fried egg,” “basket,” “bird’s eye,” hemozoin, etc.) can be “seen” by the software. The software also patches together all 800 tiled digital images to provide a zoomable image, much like a virtual microscope, thereby making human review of the entire slide possible.

### WHO 55 slide reading test

This challenge was selected because it reflects a testing standard published by the World Health Organization (WHO) [2], pp 31–40 designed to provide a defined and therefore consistent means of assessing the proficiency of malaria slide reading. Not only are the numbers of both positive and negative slides that are to make up the test proscribed, but also the densities and species composition of the positive slides. By adhering to the precepts of this test, the proficiency of individuals, even if tested in different times and places, are truly comparable. The slide set composition and accreditation standards set forth in this WHO reference are designed to evaluate clinical microscopists working as reference microscopists and/or more advanced, skilled trainers in national programs. Therefore, as opposed to the less rigorous tests that are also described in this reference (testing of ‘peripheral microscopists’), this test was chosen to be able to assess the device’s performance as if it were an experienced human reader at an advanced level of competence.

Slides from the Malaria Research and Reagent Reference Resource Center (MR4) archive comprised 49 of the 55 slide test set. The remaining six slides were selected from the National Archive of Malaria Slides (NAMS) of Equatorial Guinea. Both archives were prepared under the guidance of Hydas World Health (HWH, Hershey PA) and adhered to the slide archiving principles that they pioneered for the MR4 archive [15]. Fifty-five slides, each with a thick and thin film and marked only by a unique eight character identifier printed in both human readable and barcode format were sent to WHT for analysis. Results were reported by electronic answer sheet.

The test consisted of 40 slides which were to be qualitatively analyzed as positive or negative along with the species, if positive. Twenty of these 40 slides were true negatives and of the remaining 20, ten were *Plasmodium falciparum* infections ranging from 75 to 165,500 parasites/μL. The remaining 10 positive slides were non-*P*. falciparum and mixed species infections in the following proportions: Four were *Plasmodium vivax* with densities between 355 and 14,809 parasites/μL, two *Plasmodium malariae* with 164 and 3,275 parasites/μL and four mixed (*P*. falciparum/P. vivax) with densities between 31 K and 129 K parasites/μL (combined parasite count). In mixed species infections each species was present in excess of 40 parasites/μL. All slides were protected by cover slips, one shielding the entire thick and thin film (25x50mm) and the other covering the label (25x25mm).

**Figure 3:**
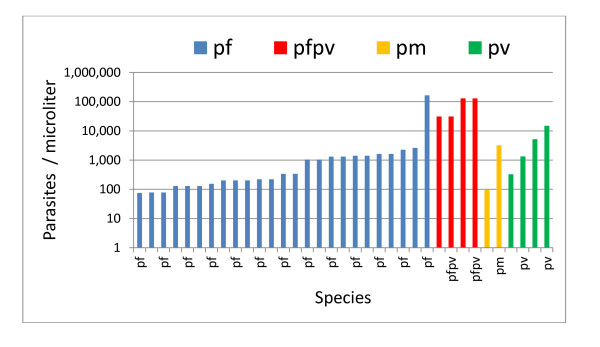
Parasitaemias of all positive slides of the WHO 55 test.

In addition to a qualitative assessment of positivity or negativity, it is important on a slide reading test to assess a diagnostician’s ability to quantify an infection. For this aspect, the WHO 55 test calls for an additional 15 slides that were marked as “counting slides.” Normally, examinees are told that these 15 slides are pf positive and that their only task is to quantify and report the severity of the infection as parasites/μL. The WHT software however, quantifies all positive slides as a matter of course. To make the evaluation truly equivalent to that of a human reader, only the reported parasitaemia for the 15 “counting” slides were used to evaluate its quantifying ability using the “level 1,2,3,4” system promulgated by the WHO. Analysis of the WHO55 test slides was carried out in the WHT facility in Columbus OH using the equipment described above. Figure 3 graphically displays the parasitaemia of all 35 positive slides used for the WHO55 test.

### Equatorial Guinea Malaria Indicator Survey (EGMIS) test

The Equatorial Guinea household based Malaria Indicator Survey is part of the Bioko Island Malaria Control Project (BIMCP) that has been implemented by Medical Care Development International (MCDI) in collaboration with the country’s National Malaria Control Programme since 2003. The BIMCP is funded by a consortium led by Marathon Oil Corporation (Houston, TX) and the Government of Equatorial Guinea. Slides collected from donors in the EGMIS were used for two reasons. First, they provided a source of blood smears from asymptomatic patients. The hypothesis that asymptomatic donors may well provide infections with a lower parasitaemia was borne out by the median of 673 parasites/μL among the 13 positive slides collected. Second, they represented slides that were prepared by the Equatoguinean technicians in the normal course of their clinical duties. The IScan Coreo Gold® scanner was transported to Equatorial Guinea, set up in a clinical laboratory and acquired images that were analysed by a laptop running the WHT software (Figure 1b). One third of the EGMIS slides were analysed by the WHT device in Malabo, demonstrating the robustness of this system and the feasibility of using it in comparable settings of the developing world.

Donors were inhabitants of Bioko Island, Equatorial Guinea, who were included in the 2010 malaria indicator survey. For the purpose of the EGMIS, the island was divided into 18 sentinel sites, with family sub-samples being randomly selected within each sentinel site area for inclusion in the study so long as they had at least one child less than 15 years of age and/or a pregnant woman currently residing in the household. Within these sampled households all children less than 15 years of age as well as pregnant women were administered malaria rapid diagnostic tests (RDTs). In addition to this RDT, a random sub-sample of these individuals also had a blood smear made that were used in this study. Further details of the Equatoguinean malaria indicator survey have been previously described [16]. In 2010 the entire survey included 2,952 households with 6,431 individuals tested for malaria.

### Sample collection methods

For the household survey, participants’ blood was collected by Equatoguinean technicians in the field by finger-prick and immediately analyzed by the ICT Rapid Diagnostic Test (ICT Malaria Combo Test, Cape Town, South Africa). The ICT RDT is a HRP-2/aldolase based immunochromatographic strip that provides results for *P. falciparum* and non- *P. falciparum* species. Since cost and logistics prevented the microscope validation of all of the nearly 6.5 thousand EGMIS samples, only a sub-sample (approximately 150) participants had microscopy slides made from their finger prick blood (one slide per individual, including both thin and thick smears made on the same slide), and a blood sample was collected on filter paper for PCR analysis if needed. The microscope slides were kept in a closed container and Giemsa-stained at the Malabo Central Hospital malaria laboratory by hospital laboratory staff within 24 hours of collection, in accordance with their Giemsa staining standard operating procedures. After staining, all slides were protected with coverslips using Poly-Mount^®^ (Polysciences, Inc., Warrington, Pennsylvania) as the mounting cement.

### Establishing expert microscopy diagnosis

Approximately two thirds of the Equatoguinean slides were scanned by the WHT device in the US and one third were scanned at the Malabo Central Hospital using the equipment described above. Ten of the original 150 slides prepared during the survey were lost or broken during preparation, WHT scanning or shipment to HWH for validation. Therefore, a total of 140 subjects had their blood subjected to the three methods of diagnosis: RDT, a computer image analysis using the system developed by WHT and classical microscopy by a panel of proven expert microscopists (“reference microscopy” carried out by Hydas World Health).

For the reference microscopy, the 140 slides were sent to a minimum of six expert microscopists, each of which provided his/her diagnosis in turn. It was this “composite microscopy diagnosis” (CMD) that served as the “gold standard” against which the WHT system was compared. HWH used a pool of 9 individuals as reference readers to validate the results for this EGMIS slide set. Six of the 9 served as reference readers for the MR4 archive project [15] and scored among the top half of the 28 readers used in that project. Two of those six have since been tested and awarded a WHO Level 1 or 2 slide reading certification. The three remaining experts served as reference readers in the creation of the WHO’s archive in Asia and also hold a Level 1 WHO slide reading certification.

The criterion was established *a priori*, that in the case of divergent reads by the microscopists, a minimum of 80% of the reads must be qualitatively concordant. Thus, more than one divergent reference read was interpreted as a slide on which the experts disagreed and a CMD was not possible. These slides were not included in the analysis. Discrepant microscope diagnoses occurred in 21 of the 140 cases, leaving 119 slides with evaluable results (Table 1).

**Table 1:** Data for 13 positive slides (above horizontal line) and for 21 slides with discordant results (below line).

**Slide #**	**WHT Result**	**WHT P/ul**	**RDT result**	**# reads agree**	**RR Dx**	**median if pos**	**# discordant reads**	**Alt Dx**	**Reference Reader COMMENTS**
63	pf	100	PF	5	pf	47	1	NEG	Thick not stained completely; Thin film: incorrectly fixed
143	pf	10,000	PF	7	pf	160	0		microfilariae present; Staining was blue color.
137	pf	1,500	PF	7	pf	596	0		Not good staining, much detritus at edge thick smear.
72	pf	1,600	PF	6	pf	652	0		Thin film incorrectly fixed
61	pf	2,800	PF	6	pf	673	0		no comments
56	Pf/pm	800	MIX	6	pf	675	0		10% ring forms; Plasmodium falciparum
118	pf	571	PF	6	pf	724	0		gametocytes (some rounded up); Thin smear: too thick + fixation inadequate
44	pf	100	PF	6	pf	1,151	0		Malaria pigment seen. Parasites not well stained. Thick film not well distributed. massive stain deposit and presence of multiple artifacts
68	Pf/pm	8,000	MIX	6	pf	1,916	0		Thick film: not completely stained; Thin film: incorrectly fixed
115	Pf/pm	31,600	MIX	6	pf	34,811	0		beautiful parasites! Slide with crack! Thick smear: irregular distribution; Thin smear: too thick
117	Pf/pm	17,200	MIX	6	pf	54,613	0		beautiful parasites! thin smear: too thick
78	pf	50	PF	4	pf	207	2	pfpo	A difficult but interesting slide. It is obviously a mixed infection of P. falciparum and either
P. vivax or P. ovale. Some parasites in the thick film have a pinkish background that I associate with P. ovale. Parasitized rbcs in the thin film lack obvious stippling and are normal in size or only very slightly enlarged. About half of the parasitized rbcs in the thin film are distorted in shape (oblong) and/or have fimbriae that is characteristic of P. ovale. This slide is designed to drive readers crazy. There are many large trophs in the thick film with a pinkish "fried egg" appearance, typical in my experience of P. ovale. There are rings with could be either ovale or falciparum. In the thick film I saw one beautiful P.f. gametocyte (confirming P falciparum) and several large, ameboid trophs in rbc's that were only slightly enlarged. This is obviously a mixed infection of falciparum and, most likely, ovale.									
74	Pf/ pm	150	MIX	3	pv	110	4	po	A difficult slide for me. It is obviously either P. vivax or P. ovale. The parasites in the thin film lack
obvious stippling and are either normal in size or only very slightly enlarged. About 50% of them however had a distorted "oblong" shape and/or fimbriae that is characteristic of P. ovale. I have called this PO because of the compactness of the trophs and stippling on the thick film but the staining on the thin film does not show the stippling or good morphology; thick film: with air bubble; pigment in WBC									
111	NEG		NEG	4	NEG		2	pf	Thick smear: irregular distribution hemolysation: staining inadequate; fixation inadequate
131	NEG		NEG	4	NEG		2	pf	contaminated with bacteria; many eosiniphils; Thin smear: too thick and grease on the slide
134	NEG		NEG	4	NEG		2	pf	Thick smear: irregular distribution; Filaria: Manzonella perstans
135	NEG		NEG	4	NEG		2	pf	Thick smear: irregular distribution Filaria: Manzonella perstans;
130	NEG		NEG	4	NEG		2	pf	Thick smear: distribution irregularFilaria! Manzonella perstans; grease on the slide
129	NEG		NEG	4	NEG		2	pf	Microfilariae of Mansonella perstans; Eosinophils ++thick smear: contaminated (bacteria + dust)
147	pf	3,000	OTRO PLAS	4	NEG		3	pf	algal contamination of stain
144	NEG		NEG	5	NEG		2	pf	not a good slide - too much precipitate; Two destroyed gamete. Too much precipitate, debris; Fixed edges
133	NEG		NEG	4	NEG		2	pf	Thick film: irregular distribution; thin film: too thin
132	NEG		MIX	4	NEG		2	pf	Thick smear: contaminated with bacteria and dust; Irregular distribution many eosinophils
128	NEG		NEG	4	NEG		2	pf	thick smear: contaminated (bacteria + dust); Thin smear: too thick
116	NEG		NEG	4	NEG		2	pf	thin smear: too thick + fixation inadequate
140	NEG		NEG	5	pf	26	2	NEG	Not good distribution of WBC in thick smear.
58	NEG		NEG	4	pf	30	2	NEG	good staining. Pf; prob neg but bc artifacts and stain deposit single parasites in thick film can’t be ruled out
126	NEG		NEG	3	pf	35	3	NEG	Thick smear: some contamination (dust)thin smear: too thick
136	NEG		NEG	3	pf	39	3	NEG	Thick smear: irregular distribution contaminated with dust; thin smear: too thick
104	NEG		NEG	3	pf	74	3	NEG	no comments
103	NEG		PF	4	pf	83	2	NEG	1x M. perstans microfilaria
43	NEG		NEG	3	pf	95	3	NEG	Many dots could be confused as chromatin but were two sizes (big and smaller). Call pf pos but many artifacts
145	pf	1,000	PF	5	pf	111	2	NEG	RINGS
142	pf	5,000	PF	5	pf	160	2	NEG	Algae present yeasts resemble Pfg

Discordant slides were not considered in the evaluation; coalesced comments from all readers in last column.

### Ethics approval

The protocol under which the blood samples were collected in Equatorial Guinea was approved by the Ethics Committee of the London School of Hygiene and Tropical Medicine (LSHT reference 5713), and by the Ethics Review Committee of the Equatorial Guinea Ministry of Health and Social Welfare. Preparation of slides for the MR4 archive was approved by the Indonesian Ministry of Health National Institute of Health Research and Development ethics review board (reference KS.02.01.2.1-4090) and the Naval Medical Research Unit #2 Institutional Review Board (reference DoD#30873). Therefore, all slides used for this test were collected from subjects covered by an ethics board approved minimal risk human use protocol and all subjects gave informed written consent in compliance with the Helsinki Declaration.

## Results

### WHO 55 slide reading test

The WHO Malaria Quality Assurance Manual uses the 55-slide core level test to score three areas: detection of parasitaemia, species identification, and parasite quantification [2]. The results are scored as straight percentages, dividing the number correct by the number possible times 100. In Table 2 the denominator used for each of the three categories is shown in the header. The WHT machine score for each parameter is shown at the bottom of Table 2. Expressed in terms of the grading scheme outlined in ref 1and Table 2, the device achieved a “Level 3” in the category of parasitaemia detection (30 correct positive/negative diagnoses out of 40 qualitative slides). For “Species Identification” it identified nine of the 20 positive slides exactly correct for a score of 45%. In only one of the 15 quantitative (counting) slides did the parasite count fall within 25% of the true median value, making the “Parasite Quantitation” score 7%. In accordance with this WHO scoring scheme, the overall score of the automated image analyser is the lowest of the three components, therefore, “Level 4.”

**Table 2:** Evaluation of WHT device on WHO55 test with respect to WHO grading scheme

**Accreditation Level**	**Detection of parasitaemia**	**Species Identification**	**Parasite Quantitation**
Based on lowest grade achieved	40 slides	20 Slides	(within 25% of true count) 15 Slides
Level 1 (Expert)	90%	90%	50%
Level 2	80% - <90%	80% - <90%	40% - <50%
Level 3	**70% - <80%**	70% - <80%	30% - <40%
Level 4	<70%	**<70%**	**<30%**
**WHT analyser** 95% Confidence Interval	**75%** CI 59 to 87	**45%** CI 23 to 68	**7%** CI 0 to 32

In addition to the WHO promulgated “Level” score however, further information can be gleaned from this WHO55 test. Using the traditional measures of sensitivity and specificity the results of the WHT automated scanning analysis on this slide set yielded a sensitivity of 80% (95% CI 56 to 94) and a specificity of 70% (95% CI 46 to 88) using only the non-counting slides (n = 40). However, since the program cannot be cognizant of the “counting slides” being pf positive, a case can be made for including those analyses in the sensitivity and specificity calculation. This raises the *n* to 55 and the resulting sensitivity increases to 89% (95% CI 73 to 97) while the specificity remains unchanged at 70%. (95% CI 46 to 88). The values used in the analysis of 40 non-counting slides only are in Table 3. The values enclosed by parentheses were used when all 55 slides are included.

**Table 3:** Sensitivity and Specificity 2x2 for WHO 55 test

		**Archive validated result (presence of parasites)**	
		Positive *n* = 20 (n = 35)	Negative *n* = 20
Machine Dx	Positive	True Positive 16 (31)	False Positive 6 (6)
	Negative	False Negative] 4 (4)	True Negative 14 (14)
		Sensitivity = 80% (89%)	Specificity = 70%

Likewise, the percentage of correctly identified species was 9, using as denominator only the 20 positive, non-counting slides to arrive at the score of 45%. By considering the 15, counting slides and not penalizing a false negative as an incorrect species, this percentage jumps to 20 correct out of a total of 32 possible or 63%; (95% CI 44 to 79).

The quantification of parasitaemia reported by the software was compared to that derived from a median value from the reference readers used to validate the slide. For each slide used in the test, a minimum of 12 reference reader valuations comprised the median value, used as the “truth” with respect to parasitaemia. As noted above and seen in Table 4 below, of the 15 counting slides, only one fell within 25% of this median parasitaemia. By performing a square root transformation, the Poisson nature of the count data distribution can be normalized. Using an alpha value of 0.01, a 99% confidence interval was determined and the interval back transformed to be expressed as count data. The interval obtained by this method did not change the number of slides deemed to be appropriately counted. A discussion of the most appropriate range to use for quantitation is beyond the scope of this paper, and for simplicity the quantitation scores of the WHT device will be calculated on the basis of the number of slides falling within either range.

**Table 4:** WHO55 quantification showing both WHO 25% range and 99% confidence interval

	**Machine**	**CMD**	**WHO**	**std**	**Confidence**	**Interval**	**Correct**
	Counts	MEDIAN	25% below	25% above	99% lower limit	99% upper limit	
Count slide 1	**5,320**	340	255	425	239	850	
Count slide 2	**3,492**	1,048	786	1,310	742	1,227	
Count slide 3	**4,480**	1,321	991	1,651	855	1,984	
Count slide 4	**2,175**	1,404	1,053	1,754	1,001	1,610	
Count slide 5	**3,081**	1,404	1,053	1,754	1,001	1,610	
Count slide 6	**7,042**	2,625	1,969	3,281	1,936	2,956	
Count slide 7	**1,608**	2,261	1,696	2,827	1,756	2,795	
Count slide 8	**250,000**	165,500	124,125	206,875	123,455	235,865	
Count slide 9	**2,100**	1,620	1,215	2,024	1,323	2,031	
Count slide10	**5,072**	340	255	425	239	850	
Count slide11	**3,096**	1,048	786	1,310	742	1,227	
Count slide12	**204**	129	97	161	92	174	
Count slide13	**192**	**154**	**116**	**193**	**79**	**199**	✔
Count slide14	**8,880**	1,321	991	1,651	855	1,984	
Count slide 15	**412**	1,620	1,215	2,024	1,323	2,031	

### EGMIS test

HWH received 140 slides from the Equatorial Guinea BIMCP Malaria Indicator Survey that had been tested both by RDT and by the WHT device. After a minimum of six reference readers had diagnosed each slide, 21 were eliminated due to discordant microscopy results. In each of these 21 discordant results, the discrepancy was between a low *P. falciparum* count seen by two or more of the readers and the remainder of the validators who reported the slide as negative (Table 1). The mean of the parasitaemia reported in the 21 discrepant cases was 75 parasites/μL (range 24 to 220). One hundred and six of the remaining 119 evaluable results were negative and 13 were positive. For two of the 13 positives cases, all reference reader diagnoses were in agreement as positive, they did not, however, agree on the species. In the first instance (slide # 78) all six readers saw pf (207 parasites/μL), however two of the six reported the presence of *Plasmodium ovale* as well (159 parasites/μL). In the case of slide # 74, three readers reported *P. vivax* (mean density 136 parasites/μL) while four others identified the parasites as *P. ovale* (mean density 77 parasites/μL). The reference readers were blinded to the reads of others and were not aware that the geographic origin of the slides was West Africa. The CMD of slides #74 and #78 was, therefore, deemed to be “Positive” in both cases, and this result was used only in the sensitivity/specificity calculations. Therefore the results reported here are predicated on 119 evaluable results, 106 were negative and 13 positives for sensitivity/specificity calculations while only 11 EGMIS positives were used in species accuracy calculations (Table 1).

**Table 5:** Parasite counting results for the 13 positives slides of the EGMIS

		**CMD**	**WHO**	**std**	**Confidence**	**Interval**	
EGMIS (+)	Machine Counts	MEDIAN	25% below	25% above	99% lower limit	99% upper limit	Correct
44	100	1,151	863	1,439	247	2,050	
**56**	**800**	**675**	**506**	**844**	**429**	**1,049**	✔
61	2,800	673	505	841	419	1,006	
63	100	47	35	59	22	96	
68	8,000	1,916	1,437	2,395	1,056	3,176	
72	1,600	652	489	815	156	1,178	
**74**	**150**	**110**	**83**	**138**	**47**	**168**	✔
**78**	**50**	**207**	**155**	**258**	**24**	**301**	✔
**115**	**31,600**	**34,811**	**26,108**	**43,514**	**24,025**	**47,726**	✔
117	17,200	54,613	40,960	68,266	38,770	75,645	
**118**	**571**	**724**	**543**	**905**	**392**	**1,225**	✔
137	1,500	596	447	745	305	1,328	
143	10,000	160	120	200	125	226	

The sensitivity and specificity of the WHT device for the analysis of these 119 evaluable EGMIS slides was 100% (95% CI 75 to 100) and 94% (95% CI 87 to 98) respectively (Table 6). The RDT results on the same specimens compared to the Reference Reader result was 100% sensitivity (95% CI 75 to 100) and 91% specificity (95% CI 83 to 95), see Table 7.

**Table 6:** Sensitivity and Specificity 2x2 for EGMIS RR results compared to WHT device

		**Reference Reader Microscopy result**	
		Positive (*n* = 13)	Negative (*n* = 106)
Machine Dx	Positive	True Positive 13	False Positive 6
	Negative	False Negative 0	True Negative 100
		Sensitivity = 100%	Specificity = 94%

**Table 7:** Sensitivity and Specificity 2x2 for EGMIS RR results compared to RDT results

		**Reference Reader Microscopy result**	
		Malaria (*n* = 13)	No Malaria (*n* = 106)
R D T Dx	Positive	True Positive 13	False Positive 10
	Negative	False Negative 0	True Negative 96
		Sensitivity = 100%	Specificity = 91%

All of the unambiguously confirmed positives were identified by the reference microscopists as pf. The WHT device correctly identified seven of the 11 as pf, while the remaining four were called mixed infections of pfpm. Therefore, a species score of 64% (95% CI 31 to 89) was achieved on the EGMIS.

With respect to quantitation of the infections, the WHT device’s parasite densities for the 11 evaluable EGMIS positive slides are shown in Table 5 above. By using either the WHO standard of within 25% of the mean (or median) or within a 99% confidence interval a total of 5 slides can be scored as correct, for a percentage of 45% (95% CI 17 to 77) quantitation score.

**Figure 4:**
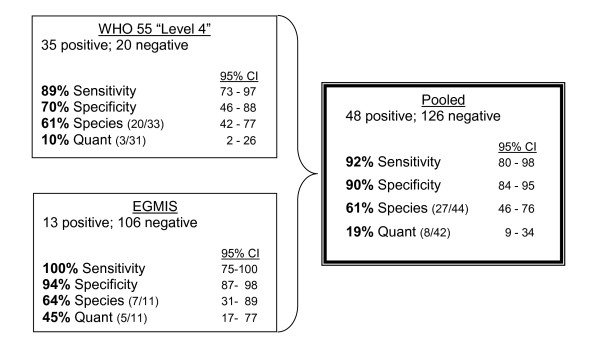
Individual and pooled test results.

The four evaluated parameters are summarized for each test in the diagram of Figure 4 below. The WHO test was also analyzed separately in order to have a basis of comparison to other WHO graded microscopy testing. This analysis yielded a “Level 4” score using the scoring methodology of the “Malaria Microscopy Quality Assurance Manual – Version 1” [2]. However, a pooling of the WHO test data with those from the EGMIS derived slides allows for the reported parameters to be calculated on a larger *n* and therefore presumably a more robust statistic.

## Discussion

Inevitably, the performance of a device in a test such as this begs the question, “how do the scores compare to existing diagnosticians.” Although difficult to answer, the increased attention to all aspects of malaria control over the past decade has provided some data with which to illuminate this question. A cross sectional survey of 17 medical treatment facilities in Kenya revealed microscope diagnosis under operational conditions to have a sensitivity and specificity of 69% and 62%, respectively [17]. A summary of the WHO55 expert testing on the African continent to date, reveals that of 101 laboratory scientists and physicians tested, 58 scored a “Level 4” with an average sensitivity and specificity of 78% and 82%, respectively [18]. The combined performance of these 101 diagnosticians with respect to the three elements evaluated by the WHO grading scheme is shown in Table 8.

**Table 8:** Evaluation of human microscopists on WHO55 test with respect to WHO standards

****Accreditation Level****	**Detection of parasitaemia**	**Species Identification**	**Parasite Quantitation**
Based on lowest grade achieved	40 slides	20 Slides	(within 25% of true count) 15 Slides
Level 1 (Expert)	90%	90%	50%
Level 2	80% - <90%	80% - <90%	40% - <50%
Level 3	70% - <80%	70% - <80%	30% - <40%
Level 4	<70%	<70%	<30%
**Composite Human (n = 101)**	**80%**	**36%**	**25%**

**Figure 5:**
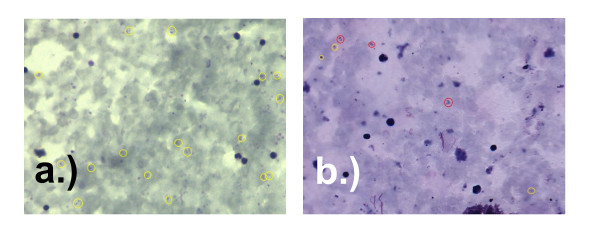
**Example of thick film image output from the WHT device annotated by the software:a.)** Yellow circles indicate parasites; **b.)** red circles are discounted parasite-like anomalies.

The performance of the WHT device to diagnose standard malaria blood smears, both thick and thin films was at least comparable to these data. For the pooled data in our evaluation, sensitivity and specificity both above 90% is noteworthy considering the fact that the device functions with minimal need for operator intervention. Despite the fact that the operators need not make the ultimate diagnosis they can, if they so desire, review any and all of the scans and view the items that the algorithm detects as malaria because the machine circles these on the stored image (Figure 5).

With only a total of 10 non-pf positives in this evaluation, the device cannot be considered to have been exhaustively tested with respect to species identification. However, having correctly identified half of them, to include 2 mixed infections, is a promising result. A sixth result was a *P. ovale* diagnosis on a *P. vivax* positive slide. These two species share many phenotypic similarities and their misidentification is an error frequently made by microscopists.

**Figure 6:**
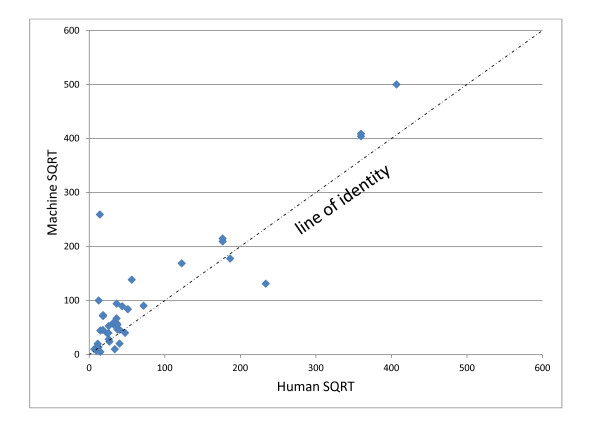
**Scatter Plot of SQRT transformation human vs machine counts,*n*** **= 42.**

**Figure 7:**
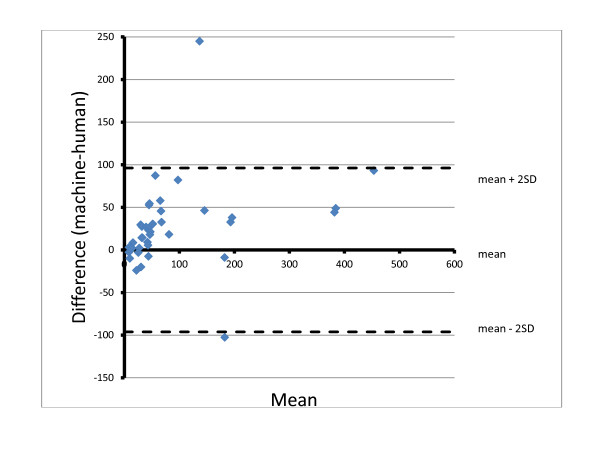
Mean-difference (Bland Altman) plot using square root transformed counts, n = 42.

The weakest parameter examined for the device was quantitation, which happens to be the most difficult aspect for most human slide readers as well, a fact evidenced by the criteria of the WHO grading scheme (Tables 2 and 8). Evaluating the results of the WHT software with respect to its performance in quantifying the infections that it found is complicated by how to determine what the appropriate range surrounding the median within which to accept a value as correct should be [19]. The WHO advocates a straight 25% margin above and below the reported mean (or median) of the archive slide. Since this approach does not take into account the variability that human reference readers exhibited for that slide, the authors favor an approach that uses a 99% confidence interval surrounding the mean, derived from the quantifications of the reference readers used to validate the archive slide. Because the counts from the device did not, for the most part, fall within either criterion, a further discussion here is moot. Figure 6 is a linear regression plotting the square root transformations of the counts of the reference readers on the abscissa against the transformed machine counts on the ordinate. Using such a transformation normalizes the Poisson distributed counting data as well as shrinks the scale to better visualize the results. Although the R^2^ value, at 0.82 indicates that the correlation to the regression line could be much better, the slope of 1.027 shows that there was a relationship between the machine and human counts in which higher parasitaemias resulted in higher machine counts. The y-intercept of 23 in the regression line is a mathematical indication that the machine tends to overestimate the parasitaemias. This finding is also evidenced by a plot of the difference between the two counts plotted on the ordinate against their mean on the abscissa also known as a Tukey mean difference, or Bland/Altman test [20]. This graph, shown in Figure 7, also uses the square roots of the counts and clearly shows most of the points to be on the positive side of the 0 line in the abscissa.

One explanation for these data is, of course, that artifacts, such as stain crystals or even platelets, are being mistakenly identified by the algorithm as parasites. The y-intercept of the regression cannot be interpreted as the projected reading for a slide with 0 parasites however, since the number of false positives was comparatively low. These data, taken together, point to the importance of the pre-scan function and its inherent threshold value.

Blood was collected on filter paper for PCR if needed, and this can still be done. However, it is felt that the benefit may not outweigh the cost and effort in this case, especially since certain conclusions can be drawn from the data as they stand. A closer examination of the 21 slides that were removed from the analysis in the EGMIS test reveals them to be slides with a very low parasitaemia (mean 75 parasites/μL, range 24–220). Only three of these were called positive by the WHT device (Table 5). Therefore, a PCR result on the 21 discarded slides (if positive, which is likely) would serve to indicate the lower limits of parasite detection. Considering the fact that the WHT device recorded four false negative results on the WHO 55 test on slides with an average parasitaemia of 140 parasites/μL, one can reasonably make the inference that the limit of reliable detection may well be in this range.

A question may be raised as to why the WHO grading scheme is not applied to the EGMIS data set. Although it would have also been scored as a “Level 4” according to those standards, it is not felt that this would not be an appropriate application of the WHO grading scheme. The EGMIS test, while comprised of 119 evaluated slides, contained only 11 positives and one species.

The Level 4 that the device scored on the WHO55 test may be somewhat misleading. Because it is the lowest level, some may interpret that to mean that it performed poorly. It should be stressed however, that this standard is designed as a test for expert readers. The multitude of species and parasitaemias presented is challenging and with only 20 positives, the scoring by percentages is unforgiving. This standard is only beginning to be applied widely enough to be able to put it into perspective by comparing the device’s score to those of human readers. The WHO55 test was included in the evaluation of the device however, because it is the only published standard that defines precisely the species and densities to use for the evaluation of malaria microscopy proficiency. Only by using slides that fit these criteria can the device’s performance be fairly and reproducibly compared to other diagnosticians, or even to future versions of itself.

The WHT analyser performed as least as well as the RDTs used on Bioko Island. Interestingly, all six of the slides falsely identified as positive by the WHT device, were also called positive by the RDT. If these slides came from subjects with a recently cleared infection, it may explain the immunological positive of the RDT, but not that of the WHT device. The scans of these specimens are being reviewed to determine what shapes triggered a positive call by the algorithm.

## Conclusions

The performance of this device during these tests where it correctly identified the presence or absence of malaria parasites 158 times out of 174 attempts (90% of cases) is noteworthy. It is also of note that this level of performance was reached without the need for highly specialized equipment. The device used standard Giemsa-stained slides and required only a personal computer and a means of digitizing the microscope derived high power fields (scanner). Although it generated 12 false positives (7% of cases), considering the multitude of artifacts that could be mistaken for parasites; we consider this also to be a promising performance. The four false negative results (3% of cases) occurred at an average parasitaemia of 140 parasites/μL which is an indication of the limit of reliable detection.

Save the operator identification of the scanning area, the system has been designed to operate without operator intervention. However, the fact that the digital scans are saved and can be reviewed is an important aspect which will allow for rapid improvement of the algorithm. An additional advantage of this system is the fact that it functions in a very analogous way to human slide readers. Unlike other devices described to date [9,10], the device uses both thick and thin films, quantifies and determines parasite species.

A few of the improvements already being worked on by the developers of this system include:

· X/Y coordinate location of parasites,

· composite images of specific species for human confirmation,

· self confirming diagnoses between parasites in thick and thin film of the same slide,

· a “confidence” value to flag those specimens that require microscopist review

· exploring the trade-off between the increased scanning time and file size versus the (presumably) improved performance (sensitivity and specificity) by using a 100x, oil immersion objective to scan the slide as opposed to the 40x ‘high, dry’ lens used in this evaluation.

The quality of malaria diagnosis and reporting is not an issue restricted to tropical countries in which the disease is endemic – among 278 US laboratory personnel surveyed, 90% reported that their labs include malaria diagnosis among their capabilities, and among those, 85% report that such diagnoses are performed in-house (it is not clear from the paper if some of the respondents were employed by the same lab, thus skewing the results) [21]. The laboratories at which these individuals work meet the six criteria of the Clinical and Laboratory Standards Institute (CSLI) to varying degrees. The criteria include slide preparation and examination protocols as well as reporting standards, but interestingly not the accuracy of the diagnoses themselves – in-house or outside slide readers are assumed to be competent without reference to QA/QC programs in place to evaluate slide reading abilities.

A study which did address proficiency test performance was a review of the American Proficiency Institute's (API) Parasitology programme. The fact that the programme sends participating laboratories three slides per year is a testament for the logistical difficulties of evaluating the microscopic diagnosis of malaria. However, irrespective of the rigor of the program, the fact that the proficiency test results over a 10-year period from 1999–2008, revealed that half of the participating laboratories failed to report a “full workup” is in itself telling. In other words, half of the laboratories failed to report either species determination, parasite quantification, or both [22].

By comparison, the WHT device has an objectively measured level of accuracy that is continually being improved to make it comparable to that of human slide readers. Its ability to scan slides rapidly would greatly assist laboratories in meeting the final three of the CSLI criteria, which include the number of thin and thick films examined and the speed at which reports can be provided to clinicians. For a first screening of clinical samples, even a relatively low specificity score, when combined with high sensitivity, results in the elimination from further consideration of the many correctly identified negative specimens, allowing technicians to concentrate on those the device marks as positive. For laboratories in non-endemic countries, such as North America, Europe or Australia, where the preponderance of slides will in fact be negative, the work saved by such screening would be significant. Further, the use of an automated diagnostic device would eliminate the uncertainties currently surrounding the question of diagnostic proficiency, which is extremely difficult to assess due to logistics and the limited availability of standardized slide sets.

## Abbreviations

BIMCP, Bioko Island Malaria Control Project; CI, Confidence interval; CSLI, Clinical and Laboratory Standards Institute; CMD, Composite microscopy diagnosis”; EGMIS, Equatorial Guinea Malaria Indicator Survey; HWH, Hydas World Health; LSHT, London School of Hygiene and Tropical Medicine; MR4, Malaria Research and Reagent Reference Resource Center; MCDI, Medical Care Development International; μL, microliter; NAMS, National Archive of Malaria Slides; PCR, Polymerase Chain Reaction; QBC, qualitative buffy coat microscopy; RBC, Red blood cells; RR, Reference Reader; STARD, Standards for Reporting of Diagnostic Accuracy; WBCs, White blood cells; WHO, World Health Organization; WHT, World Health Technology.

## Competing interests

MP and JB are the inventors and developers of the device that has been tested in this publication and therefore may not be considered to be unbiased. They supplied not only the equipment and their programs but also contributed to the methods section of this paper to describe the workings of the system. The remaining authors have no commercial interest in the findings of this study.

## Authors’ contributions

WP and RJ conceived of the study, analyzed the data and prepared the manuscript. VC contributed to data analysis. MG, IK and CS reviewed the manuscript and provided valuable suggestions toward data analysis. MM and MT coordinated the collection and preparation of all slides for the EGMIS portion of the study as well as that of the Equatoguinean archive slides that contributed to the WHO55 sets. All authors read and approved the final manuscript.

## Authors’ information

WP and RJ work for Hydas World Health (HWH), a non-profit which initiated the concept of National Archives of Malaria Slides (NAMS) and, with the US Navy’s NAMRU-2 overseas laboratory, created the proof-of-concept MR4 archive, the use of which is administered by NIH. To date, HWH has been involved in the creation of NAMS in Equatorial Guinea and Ghana, and is beginning their creation in Ethiopia and Zambia.
